# Wealth inequality and utilization of reproductive health services in the Republic of Vanuatu: insights from the multiple indicator cluster survey, 2007

**DOI:** 10.1186/1475-9276-10-58

**Published:** 2011-12-02

**Authors:** Mosiur Rahman, Syed E Haque, Md G Mostofa, Len Tarivonda, Muhammad Shuaib

**Affiliations:** 1 Department of Population Science and Human Resource Development, University of Rajshahi, Rajshahi-6205, Bangladesh; 2 Department of International Health, Division of Public Health, Tokyo Medical and Dental University, Tokyo, Japan; 3 Department of Community and Global Health, the University of Tokyo, Japan; 4 Department of Population Science and Human Resource Development, University of Rajshahi, Rajshahi-6205, Bangladesh; 5 Department of Public Health, Ministry of Health, Republic of Vanuatu; 6 Institute of Statistical Research and Training, University of Dhaka, Bangladesh

**Keywords:** inequalities, ante-natal care, institutional deliveries, counseling and testing HIV/AIDS

## Abstract

**Background:**

Although the Republic of Vanuatu has improved maternal indicators, more needs to be done to improve equity among the poorest in the use of reproductive health services to expedite the progress towards the Millennium Development Goal 5(MDG 5) target. While large developing country studies provide evidence of a rich-poor gap in reproductive health services utilization, not much is written in terms of Pacific Islands. Thus, this study aims to examine the degree of inequality in utilization of reproductive health services in a nationally representative sample of Vanuatu households.

**Methods:**

This paper used data from the 2007 Vanuatu Multiple Indicator Cluster Survey (MICS). The analyses were based on responses from 615 ever married women, living with at least one child below two years of age. Outcomes included antenatal care (ANC) and use of birth attendants at delivery, place of delivery, and counseling and testing for HIV/AIDS. Descriptive statistics and multivariate logistic regression methods were employed in the analysis.

**Results:**

Findings revealed that the economic well-being status of the household to which women belong, played a crucial role in explaining the variation in service utilization. Inequality in utilization was found to be more pronounced between the poorest and richest groups within the wealth quintiles. In adjusted models, mothers in the richest bands of wealth were 5.50 (95% confidence interval [CI]: 1.34-22.47), 2.12 (95% CI: 1.02-3.42), 4.0 (95% CI 1.58-10.10), and 2.0 (95% CI 1.02-5.88) times more likely to have assisted delivery from medically trained personnel, have institutional deliveries, and have counseling and testing for HIV/AIDS.

**Conclusions:**

Association between household wealth inequality and utilization of ANC and delivery assistance from medically trained personnel, institutional delivery, and counseling and testing for HIV/AIDS suggest that higher utilization of reproductive health care services in Vanuatu poor-rich inequalities need to be addressed. Reducing poverty and making services more available and accessible to the poor may be essential for improving overall reproductive health care utilization rate in Vanuatu.

## Introduction

Despite substantial progress achieved in improving maternal health status in the Pacific regions in recent decades, maternal health remains a serious concern across the region [1]. The leading causes of maternal death are similar to those reported globally: postpartum hemorrhage, preeclampsia, obstructed labor, puerperal sepsis, and complications of unsafe abortion [1]. Vanuatu is an archipelagic nation of 83 islands and is the third poorest country in the Pacific region with a per capita GDP of less than US$ 1,276 [2]. The 2009 National Census of Population and Housing reported that the population of Vanuatu to be 234,023, with a growth rate of 2.3% per annum [3]. The fertility rate in Vanuatu is high, with each woman bearing an average of five to six children [4]. Vanuatu is on track to meet some of the MDGs including promoting gender equality, empowering women (MDG 3) and reducing child mortality (MDG 4). Despite this, maternal mortality ratio (MMR) in Vanuatu is still high. According to the recent statistics of Asian Development Bank (ADB), MMR in Vanuatu was 130/100,000 live-births [2]. To meet the goal of MDG5, Vanuatu has to reduce the MMR up to 24/100,000 live births by the year 2015 [4].

There is a general consensus that the use of maternal health services reduces MMR and improves the reproductive health of women [5]. Contact with a medically trained provider during the antenatal and delivery period in particular provides an opportunity for delivery of numerous proven interventions to improve the health and survival of the mother and newborn [6]. The Ministry of Health of Vanuatu estimated that about 20% of births took place outside health facilities (home delivery) in 2007, where, in most cases, a traditional birth attendant (TBA) assisted the delivery [7]. Nationally, in 2007, 84% of mothers reported receiving at least one episode of antenatal care (ANC) from a skilled health professional: a doctor, midwife or nurse. This rate was higher in the urban areas at 87% and lower in the rural areas at 84% [7]. Although the country has improved maternal and child indicators and other MDGs, equal access to health care has been viewed as one of the prime concern towards ensuring 'Health for all' and more needs to be done to improve equity among the poorest. Therefore, it is necessary to analyze the situation using the equity lens and endeavor towards reaching those who are lagging behind in terms of the health outcomes and uptake of essential maternal health interventions.

Evidence around the world suggests that being poor is positively correlated with poorer health status and negative health outcomes. Much of it responsible for poorer uptake of preventive, promotive and curative aspects of health care services by these groups of people belonging to the lower economic strata [8-15]. Findings from the Demographic and Health Surveys (DHS) conducted in 56 countries in Africa, Asia, and Latin America between 1990-2002 showed that the poorest were less likely than the wealthiest to use basic health services such as immunization, maternity care, and family planning. On average, births to women in the richest quintile were nearly five times more likely to be attended by a trained professional such as a doctor, nurse, or midwife [16]. In Indonesia, impact research found that village midwifery services reached mostly the rich, who could afford to pay, but left the poor still unable to access skilled care [17]. Findings from Ghana showed another rich-poor gap-wealthier households benefited more from the delivery fee-exemption policy through decreased costs of delivery [18]. Public provisioning of health care services across the developing world has provoked questions regarding identifying the proper beneficiaries as the target of public subsidies and carefully oriented policy measures aimed at ensuring equal access and use of health services among these disadvantaged population groups. Women and children among the poor are more vulnerable in terms of access to health care compared to adult men [14]. In other words, maternal and child health care services are more likely to demonstrate sharp inequality in utilization. Developing countries in the Asia-Pacific region are in different stages of economic development and have varying levels of health systems. For the very poor, outreach of adequate basic health services is still a challenge [19]. The income of the poorest 20% of households in Vanuatu was 2% of the total income of all households, demonstrating the two extremes in Vanuatu with a vast difference between high and low income households [7]. However, socio economic differences in Vanuatu, particularly rich-poor gaps, in health care consumption are large and often quite resilient, indicating that services are not reaching all population groups equally [19].

While large developing country studies provide evidence of a rich-poor gap in reproductive health services utilization [20-25], not much is written in terms of Pacific Islands. This paper therefore, makes an attempt to examine the degree of inequality in utilization of preventive aspects of maternal and child health in Vanuatu that pose significant barriers to maternal health care access and utilization, and thereby impact maternal mortality. The exercise is primarily oriented towards identifying the magnitude of economic inequality based on a wealth index. This ensures continued improvement in reproductive health service utilization in Vanuatu and in Pacific region in general.

## Methods

### Data sources

**Figure 1:**
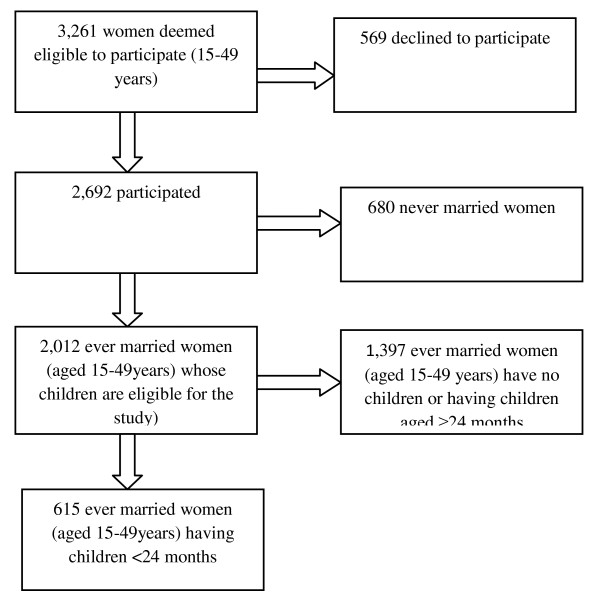
**Selection of the sample**.

The present study used data from the 2007 Vanuatu Multiple Indicator Cluster Survey (MICS), conducted by the Ministry of Health Government of Vanuatu and technical support was provided by the UNICEF and the Global Fund from November 01 to December 20, 2007. The sample for MICS Vanuatu-2007 is a probability-based, stratified cluster sample of 3000 households. They were selected in 120 clusters, each of size 25 households. The sample was selected in two stages. The first stage consisted of first stratifying the PSUs by province and within-province by urban/rural in two provinces (namely Shefa and Sanma) and then selecting 120 PSUs with probability proportionate to size or pps. At the second-stage, a fixed sample size of exactly 25 households was selected from each PSU, using systematic, equal-probability sampling or epsem. Thus a total of 3000 households were selected (120 clusters time's 25 households). The PSUs were borrowed from a sampling frame created for the 1999 Population Census of Vanuatu, and were termed enumeration areas (EA). The 2007 MICS Vanuatu used three questionnaires. Of the 3,261 women (aged 15-49 years) deemed eligible to participate in the women's questionnaire of maternal and child health behaviors and outcomes, 2,692 participated (83.0% response rate). The present analyses included only those ever married women (aged 15-49 years) having at least one child below two years of age (615 unweighted sample size; weighted value is 685) (Figure 1).

### Outcome measures

To assess the assessment of reproductive health service utilization we analyzed five outcome variables: ANC and delivery assistance provider qualification, place of delivery, and counseling and testing for HIV/AIDS. To assess the utilization of ANC services and delivery assistance according to the type of provider, a variable for type of provider was constructed from combined responses to a question whether the respondent had obtained any advice/treatment, with responses to a follow-up question on the particular practitioner consulted. Responses to these questions were organized into two categories: whether the women obtained services from (1) a medically trained provider (e.g., i.e., doctor, nurse, or midwife); or whether; (2) received services from a non-medically trained provider (traditional birth attendants, community health workers, relatives, others, and no care). A binary variable was also created to asses respondents' place of delivery (institutional e.g., i.e., government/private health centers or non-institutional e.g., i.e., respondents or others home). Besides, two binary variables were also created to asses counseling and testing for HIV/AIDS.

### Wealth index

The household wealth index is used as a proxy indicator for household wealth status in this analysis. The 2007 MICS Vanuatu wealth index was originally constructed using the entire sample from the MICS on existing data on a household's ownership of selected assets [26]. Principal components analysis was performed by using information on the ownership of household goods and amenities (assets) to assign weights to each household asset, and obtain wealth scores for each household in the sample (the assets or variables used in these calculations were as follows: [number of persons per sleeping room; type of floor; type of roof; type of wall; type of cooking fuel; presence of household assets including electricity supply, radio, TV, mobile phone, static phone, refrigerator, watch, bicycle, motorcycle, cart, car, motorized boat and canoe; source of drinking water; and, type of sanitary facility]). Each household was then weighted by the number of household members, and the household population was divided into five groups of equal size, from the poorest quintile to the richest quintile, based on the wealth scores of households they were living in. The wealth index is assumed to capture the underlying long-term wealth through information on the household assets, and is intended to produce a ranking of households by wealth, from poorest to richest [27].

### Covariates

We included several socio-demographic variables theoretically and empirically linked to maternal health services utilization [28-30]. We classified maternal current age into empirically important groups (younger 15-24 years, middle age 25-34 years, or older age 35-49 years) [31]. Education of the woman was defined in terms of the formal education system of Vanuatu: no education (0 year), primary (1-5 years), or secondary and higher (6 years or more). Place of residence was categorized as rural vs. urban. Parity was categorized as one, two, or three or more. Total numbers of household members were classified in tertiles (2-4, 5-6, or 7+). Mother's tongue of head was categorized as Bislama or others, and religion of head as Christian or others. However, continuous variables such as age, education, and total number of household members were proved to provide non-linear relationship, therefore these variables were divided into a number of segments of an equal width, and we treated these new variables as categorical variables.

### Statistical analyses

We calculated descriptive statistics for demographic, socio-economic, and reproductive health service utilization characteristics for our sample. Rate-ratios were calculated in measuring inequities. However, since rate-ratios only take into account the two extreme socio-economic groups-in this case wealth quintiles 1 and 5-the wealth quintiles in the middle i.e., 2, 3 and 4 are disregarded. Hence rate-ratios do not give a composite measure of inequality.

To rectify this flaw of rate-ratios, concentration curves were constructed, using an Ms-Excel spreadsheet. The concentration curve plots the cumulative percentage of the health variable (yaxis) against the cumulative percentage of the population, ranked by living standards, beginning with the poorest, and ending with the richest (x-axis). In other words, it plots shares of the health variable against quintiles of the living standards variable. In other words, concentration curves capture the use of health interventions, cumulatively for each wealth quintile [32]. A concentration curve that lies below the diagonal line signifies the presence of inequities favoring the rich. When the concentration curve lies above the line of equality, there is inequity favoring the poor. The degree of inequity increases when the concentration curve is further from the line of equality [20]. Concentration curves are a good graphical illustration to identify whether socioeconomic inequality in some health sector variable exists and whether it is more pronounced at one point in time than another (or in one country than another).

We created 5 fully adjusted models to analyze the appropriate binary of each health services utilization outcome. For the logistic regression analysis, all variables were entered in one step (fitting the model using the ''enter'' criteria in SPSS for Windows 16.0). Adjusted odds ratios with their 95% confidence intervals (CI) were then calculated. Odds ratios were estimated to see the strength of the associations while 95% confidence intervals were estimated for significance testing. Multicolinearity in the logistic regression analyses in our study was checked by examining the standard errors for the regression coefficients. A standard error larger than 2.0 indicates numerical problems, such as multicollinearity among the independent variables [33]. However, in our study all of the independent variables in all five fitted models for each health outcome variable had a standard error < 0.90, indicating an absence of multicolinearity. All analyses were weighted; SPSS, version 16.0 and was used for all analyses.

### Ethical considerations

The study is based on secondary data analyses of existing publicly available survey data with all identifying information removed. The survey obtained informed consent from the mothers included in the study before asking any questions.

## Results

### Descriptive statistics

More than two-fifths of the women (44.4%) were 25-34 years old, 63.3% were married for the first time below the age of 15 years. Approximately two-thirds of the mothers (63.2 completed primary level of education and 9.5% of the head's mother tongue was Bislama. Over three quarters of mothers lived in rural areas and 10.7% mothers were in the richest bands of wealth (Table 1).

**Table 1:** Descriptive Demographic and Wealth Characteristics for Ever Married Mothers in the 2007 Vanuatu Multiple Indicator Cluster Survey (N = 615)

Characteristics	Number of cases (n)^†^	Percentage (%) ^††^
**Maternal age**		
15-24 y	218	36.6
25-34 y	279	44.4
35-49 y	118	19.0
		
**Mother's age at first marriage**		
< 18 y	202	36.7
18+ y	413	63.3
		
**Maternal education**		
No education	54	8.9
Primary	366	63.2
Secondary and higher	195	27.9
		
**Mother tongue of head**		
Bislama	105	9.5
Other Language	510	90.5
		
**Parity**		
1	150	23.0
2	245	40.7
3+	220	36.4
		
**Household members (tertile)**		
2-4	195	33.3
5-6	216	35.1
7+	204	31.6
		
**Residence**		
Rural	412	84.3
Urban	203	15.7
		
**Religion of head**		
Christian	584	94.4
Others	31	5.6
		
**Wealth index**		
Poor	128	25.6
Second	128	26.5
Middle	106	18.8
Fourth	127	18.4
Richest	126	10.7

^† ^Unweighted n's and †† weighted percentages (%).

A total of 84.0% sampled mothers received ANC from medically trained providers. About 80.2% deliveries took place at government or private health care facilities. 73.7% were assisted by medically trained providers. Only 29.0% mothers reported receiving AIDS counseling, and 11.8% mothers tested for HIV/AIDs (Table 1).

The rich-poor rate ratios for the various reproductive services are given in Table 2. The rate-ratios that are more than one indicate that those interventions are used more by rich women than the poorer ones. For example, a rate-ratio of 1.67 for delivery assistance by MTP indicates that women in the richest bands of wealth were delivered by skilled providers 67% more than their poorest counterparts. The same is true for receiving ANC from skilled providers, for institutional deliveries, and for receiving counseling and testing for HIV/AIDs (Table 2).

**Table 2:** Rate-ratios for Health Care Utilization by Wealth Quintile for Ever Married Mothers in the 2007 Vanuatu Multiple Indicator Cluster Survey (N = 615)

Measure	Utilization rate	Rate-ratio (rich/poor)
**ANC from MTP**	84.0	1.14
**Delivery assistance by MTP**	73.7	1.67
**Institutional delivery**	80.2	1.41
**Counseling about AIDs or the AIDs virus**	29.0	2.56
**Testing for HIV/AIDs**	11.8	3.34

**Figure 2:**
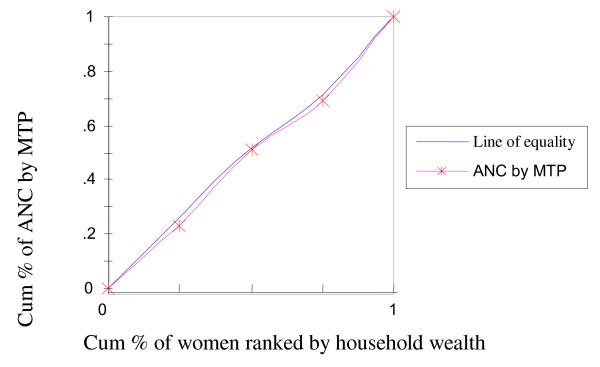
**ANC by medically trained provider (MTP)**.

**Figure 3:**
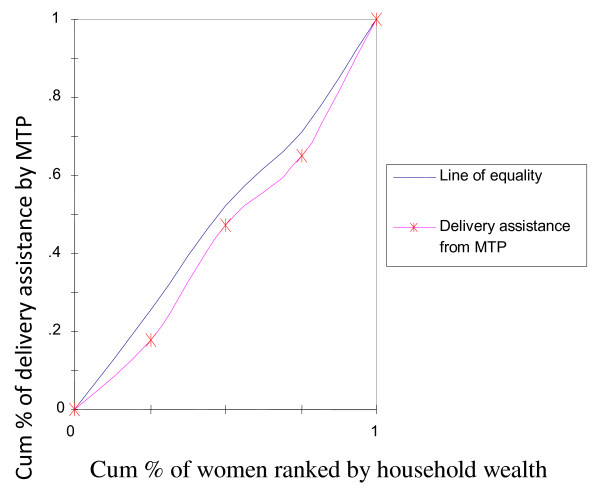
**Delivery assistance from MTP**.

**Figure 4:**
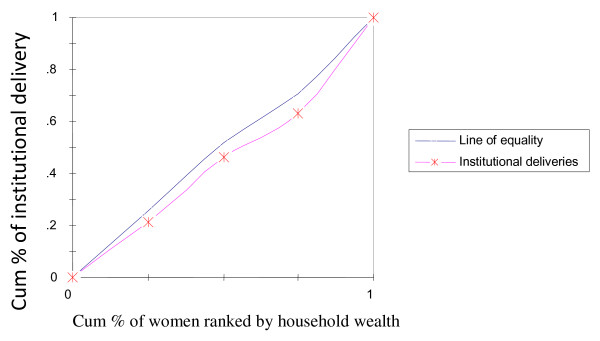
**Place of delivery (institutional)**.

**Figure 5:**
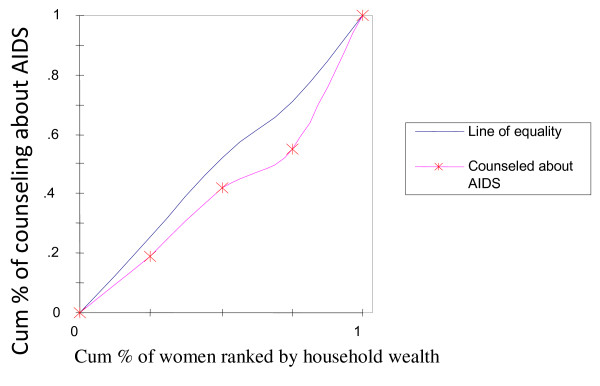
**Counseling about AIDS or AIDS virus**.

**Figure 6:**
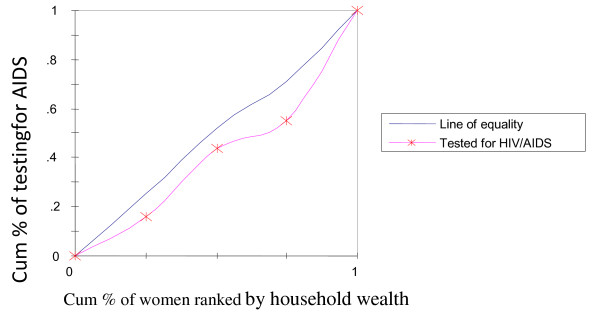
**Testing for HIV/AIDS**.

The concentration curve for receiving ANC from medically trained providers (Figure 2) had a pro-rich bias; it was more concentrated among the wealthy. Similar scenarios were depicted in Figure 3 and 4 with respect to institutional deliveries and assisted deliverers from medically trained providers, i.e., inequity with a pro-rich bias where institutional deliveries and assisted deliveries by MTP were more prevalent among the wealthy. Figure 5 and 6, showed that counseling and testing for HIV/AIDs was seen to be highly inequitable and to the advantage of the rich.

### Association between wealth quintile and reproductive health services utilization

Mothers in the second category of wealth band (adjusted odds ratio [AOR]: 1.98; 95% confidence interval [CI]: 1.06-3.68) were associated with receiving ANC from medically trained providers (Table 3). Mothers in the second (AOR: 2.93; 95% CI: 1.63-5.25), middle (AOR: 2.19; 95% CI: 1.17-4.09), fourth (AOR: 4.17; 95% CI: 1.17-4.09), and richest (AOR: 5.50; 95% CI: 1.34-22.47) quintiles of the wealth index were associated with delivery assistance from medically trained providers. Significant association was found between mothers in the richest (AOR: 2.12; 95% CI: 1.02-3.42) bands of wealth and institutional deliveries. Mothers in the richest bands of wealth were also found to be associated with HIV/AIDs counseling (AOR: 4.00; 95% CI: 1.58-10.10) and testing for HIV/AIDs (AOR: 2.00; 95% CI: 1.02-5.88) (Table 3).

**Table 3:** Adjusted ORs and 95% CIs for Associations between Aspects of Wealth Inequality and other Covariates and Health Care Utilization for Ever Married Mothers in the 2007 Vanuatu Multiple Indicator Cluster Survey (N = 615)

Variables	AOR (95% CI)				
	
	Type of provider for ANC (MTP)	Type of provider for delivery assistance (MTP)	Place of delivery (institutional)	Counseling about AIDS or the AIDS virus	Testing for HIV/AIDS
**Wealth index**					
Poor	1.00	1.00	1.00	1.00	1.00
Second	1.98 (1.06-3.68)	2.93 (1.63-5.25)	1.82 (0.80-3.09)	1.08 (0.63-1.85)	1.92 (0.86-4.26)
Middle	1.18 (0.63-2.19)	2.19 (1.17-4.09)	1.57 (0.47-4.09)	0.87 (0.47-1.60)	0.96 (0.37-2.53)
Fourth	1.80 (0.82-3.96)	4.17 (1.94-8.94)	1.95 (0.89-4.23)	2.11 (1.15-3.84)	1.77 (0.71-4.48)
Richest	1.04 (0.31-3.56)	5.50 (1.34-22.47)	2.12 (1.02-3.42)	4.00 (1.58-10.10)	2.00 (1.02-5.88)
**Maternal age**					
15-24 y	1.00	1.00	1.00	1.00	1.00
25-34 y	1.21 (0.67-2.17)	1.08 (0.61-1.93)	1.12 (0.59-2.13)	0.79 (0.49-1.25)	1.31 (0.71-2.43)
35-49 y	0.76 (0.34-1.71)	1.34 (0.58-3.12)	0.75 (0.31-1.80)	0.86 (0.44-1.70)	1.41 (0.53-3.69)
**Mother's age at first marriage**					
< 18 y	1.00	1.00	1.00		1.00
18+ y	0.86 (0.53-1.40)	1.18 (0.73-1.89)	1.22 (0.73-2.05)		0.69 (0.40-1.16)
**Maternal education**					
No education	1.00	1.00	1.00	1.00	1.00
Primary	0.92 (0.42-2.02)	1.09 (0.50-2.28)	1.01 (0.44-2.31)	2.54 (0.92-7.02)	1.94 (0.54-7.04)
Secondary and higher	2.24 (1.00-5.83)	2.08 (1.01-5.23)	1.39 (0.52-3.67)	3.53 (1.36-9.16)	2.94 (1.03-11.44)
**Mother tongue of head**					
Bislama	1.00	1.00	1.00	1.00	1.00
Others	1.23 (0.53-2.86)	2.81 (1.17-6.78)	1.75 (0.67-4.57)	1.61 (0.82-3.17)	2.39 (0.95-6.01)
**Parity**					
1	1.00	1.00	1.00	1.00	1.00
2	0.70 (0.36-1.37)	1.09 (0.58-2.06)	0.96 (0.47-1.97)	1.00 (0.59-1.69)	0.71 (0.36-1.39)
3+	0.84 (0.36-1.95)	0.55 (0.24-1.24)	0.62 (0.25-1.54)	1.11 (0.56-2.17)	0.45 (0.18-1.12)
**Household members (tertile)**					
2-4	1.00	1.00	1.00	1.00	1.00
5-6	1.04 (0.58-1.87)	1.18 (0.65-2.13)	0.93 (0.48-1.79)	1.51 (0.91-2.46)	0.73 (0.36-1.47)
7+	0.91 (0.48-1.72)	1.12 (0.59-2.15)	0.69 (0.34-1.39)	2.01 (1.21-3.32)	1.65 (0.87-3.13)
**Residence**					
Rural	1.00	1.00	1.00	1.00	1.00
Urban	0.56 (0.30-2.37)	1.57 (0.51-4.81)	2.55 (0.72-9.05)	1.21 (0.59-2.50)	2.28 (1.01-5.47)
**Religion of head**					
Christian	1.00	1.00	1.00	1.00	1.00
Others	0.43 (0.17-0.97)	0.78 (0.28-2.19)	0.32 (0.11-0.90)	4.04 (1.58-10.34)	6.37 (2.14-18.91)
**ANC provider qualification**					
NMTP	----	1.00	1.00	1.00	1.00
MTP		11.03 (9.29-17.38)	14.09 (8.31-23.90)	1.90 (1.01-3.80)	4.17 (1.28-13.53)
**Delivery assistance**					
NMTP	----	----	**----**	1.00	1.00
MTP				14.09 (8.31-23.90)	1.13 (0.52-2.39)
**Counseling about AIDS or the AIDS virus**					
No	1.00	1.00	1.00	**---**	1.00
Yes	2.50 (1.28-4.89)	3.30 (1.68-6.48)	1.77 (1.01-3.48)		20.80 (19.32-53.48)
**Testing for HIV/AIDS**					
No	1.00	1.00	1.00	1.00	**-----**
Yes	2.23 (0.67-7.48)	1.48 (0.67-2.48)	1.60 (0.52-4.87)	18.80 (16.32-43.48)	

MTP: medically trained personnel.

NMTP: Non-medically trained personnel.

### Reproductive health services utilization and other covariates

Maternal education (secondary and higher), religion (others) and counseling about HIV/AIDs (yes) were found to be associated with receiving ANC from medically trained providers (Table 3). Significant association was found among delivery assistance from medically trained providers and maternal education (secondary and higher), mother tongues of head (others), receiving ANC (from medically trained providers), and HIV/AIDs counseling (yes). Religion of head (others), receiving ANC (from medically trained providers), and HIV/AIDs counseling was found to be associated with institutional deliveries. Maternal education (secondary and higher), religion of head (other), receiving ANC (from medically trained providers), and delivery assistance (from medically trained providers) was found to be associated with counseling and testing for HIV/AIDs. In addition, significant association was found between HIV/AIDs counseling and household members (7+) (Table 3).

## Discussion

This paper attempted to examine inequalities in terms of economic well being in the preventive care of reproductive health in the Republic of Vanuatu based on a wealth index constructed from available information. The findings reveal that the economic well-being status of the household to which a woman belongs to plays a crucial role in explaining the variation in service utilization. Inequality in utilization was found to be more pronounced between the poorest and richest groups within the wealth quintiles.

The results of this study provide evidence of the positive association between mothers in the richest bands of wealth and assisted delivery from medically trained personnel, institutional deliveries, and counseling and testing for HIV/AIDS. Mothers in the second category of wealth quintile were also found to be associated receiving ANC from medically trained personnel. This is independent of maternal age, mother's education attainment, place of residence, parity, and other important factors. Our finding of the association of richest bands of wealth with the important indicators of reproductive health service utilization is consistent with the findings of previous studies in other developing countries [20,23,34,35] and provides further evidence that wealth inequality is an important risk factor for lower reproductive health service utilization in the Republic of Vanuatu.

Addressing the poor-rich inequalities in maternity care is essential for achieving the MDGs for maternal health in the Republic of Vanuatu. All health facilities and aid posts charge fees in Vanuatu. Average out-of-pocket costs in 2005 were $14 per capita per year, or $6 if payments to private providers are excluded [36]. When adding on indirect costs that the women and those accompanying them are likely to incur (e.g. transport cost), payment for deliveries could be a barrier to use of reproductive health services by trained providers for those in the poorest segments of the population. It is therefore worthwhile to revisit the policy of charging women for reproductive health services and possibly make blanket exemptions in those regions where the poverty levels/HDI are the lowest in order to increase uptake of interventions caused by demand side factors.

As expected, maternal secondary and higher education had a positive association with receiving ANC and delivery assistance from medically trained personnel and counseling and testing for HIV/AIDS. Numerous studies conducted in the developing countries over the last decade also showed a nearly universal, positive association between maternal education and the utilization of reproductive health services, a relation that has persisted in many societies even when the household's socioeconomic status has been held constant [37-39].

It was expected that ANC visits would have a positive impact on the utilization of reproductive health services [40,41]. It has been suggested one of the best things that ANC could accomplish would be to influence women to select a trained attendant during and after delivery [42]. The present authors found that compared to those who received no ANC from medically trained personnel, the odds of a mother who received delivery assistance from medically trained personnel were 11.03 times higher, while the odds of counseling and tested for HIV/AIDS were 1.90 and 4.17 times higher. In this study, mother's tongue of the head was found to be an important determinant of assisted deliveries from medically trained personnel.

### Strengths and limitations

The main strength of this study is that the data came from a large nationally representative survey carried out in 2007. A relevant subset was extracted consisting of ever-married females aged 15-49 years who had a live birth in the 2 years preceding the survey, giving a large unweighted sample size of 615. The interviewers of this survey were trained to respond to questions about the selected topics, and fieldwork was monitored through visits by representatives from MOH and UNICEF.

Some limitations should be considered when interpreting our findings. First, the current analyses are cross sectional that involves reporting of past behaviors and therefore a possible chance of recall bias. However a 2-year recall period was chosen to minimize recall bias. Second, the study was based on self-reported outcomes and might have caused a response bias. However, MICS stated that respondents were informed about the importance of their giving accurate responses and also assured the confidentiality of their responses. Third, the study can be criticized for using an indirect measure of household wealth. However, due to the fact that in developing countries like Vanuatu it is hard to obtain reliable income and expenditure data, an asset-based index is generally considered a good proxy for household wealth status. Fourth, although our study found that the poorest group is significantly different from the richest group in the expected direction in reproductive health service utilization, with the exception of 'type of provider for delivery assistance,' there are generally insignificant differences among other groups, although the direction is generally positive. It could be that five categories for the wealth index are is many given the number of cases here. Maybe a four or three category variable would be better and provide a more significant result. Finally, this study was based on a survey of women that was conducted up to two years after the birth of a child. There may be women that died after giving birth but would not make it as part of the study because they were deceased by the time the interview would take place. The study therefore misses these potential cases. Longitudinal research design where women are followed from time of birth onward is needed to provide clarity regarding these concerns. Despite these limitations the results have elicited important information that could serve as a basis for future planning to improve the utilization of reproductive services among women in Vanuatu.

## Conclusion

In conclusion, household wealth status was associated with the higher utilization of reproductive health care services among women in the Republic of Vanuatu. In interventions aimed at improving reproductive health service utilization among women in Vanuatu, poor-rich inequalities need to be addressed. In general, the message is clear in terms of reproductive health service utilizations and its relationship with poverty. These findings may be relevant in other resource limited settings of pacific regions as well as where the rate of utilization of reproductive health services is lower.

## Competing interests

The authors declare that they have no competing interests.

## Authors' contributions

MR originated the study and contributed to the study design, statistical analysis, and the writing of the article. SEH helped to conceptualize the study and contributed to the study design; interpretation of the data, and revisions to the article. MGM and LT contributed to the interpretation of data and to revisions to the article. MS monitored study progress, contributed to the conception and design of the study, and critically revised the article. All authors read and approved the final manuscript.

## References

[B1] World health Organization (WHO) Goal 5-Improve maternal health: Pacific Islands Regional MDG Report Geneva 2004

[B2] Asian Development Bank (ADB) Vanuatu Millennium Development Goals http://www.adb.org/Documents/Fact_Sheets/VAN.pdf (accessed on January 2011)

[B3] World Health Organization (WHO) Western Pacific Region Country profiles http://www.wpro.who.int/countries/van/(accessed on January 20, 2011)

[B4] AUSAID Vanuatu country overview 2010 Australian Government

[B5] Munsur AM Atia A Kawahara K Relationship between educational attainment and maternal health care utilization in Bangladesh: evidence from the 2005 Bangladesh household income and expenditure survey Research Journal of Medical sciences 2010 4 1 33 37

[B6] Countdown to 2015 decade report 2000-2010 Taking stock of maternal, newborn and child survival 2010 Geneva: World Health Organization and UNICEF 10.1016/S0140-6736(10)60678-2 20569843

[B7] Ministry of Health (MOH) Millennium development Goals 2010 Report for Vanuatu Prime Minister's Office 2010 The Republic of Vanuatu22135594

[B8] Magadi MA Madise Rodrigues Frequency and timing of antenatal care in Kenya: Explaining variations between women of different communities Social Science and Medicine 2000 5 4 551 561 10.1016/s0277-9536(99)00495-5 10868670

[B9] Bhatia J Cleland Determinants of maternal care in a region of South India Health Transition Review 1995 6 45 60

[B10] Population Refrenece Bureau (PRB) Improving the health of the World's poorest people Washington, DC 2004

[B11] Wagstaff Adam Watanabe Naoko Socioeconomic Inequalities in Child Malnutrition in the Developing World http://elibrary.worldbank.org/content/workingpaper/10.1596/1813-9450-2434 on April 15, 2003

[B12] Whitehead Margaret Timothy Evans et al "Developing the policy response to inequities in health: a global perspective," in challenging inequities in health: from ethics to action 2001 New York: Oxford University Press 314 15

[B13] Gwatkin Davidson The current state of knowledge about targeting health programs to reach the poor http://siteresources.worldbank.org/INTPAH/Resources/Publications/Recent-Papers/targeting.pdf on June 3, 2003

[B14] Mahmud S How equitable is access to and use of reproductive health care and family planning services in Bangladesh? A review of the evidence Paper presented at the IUSSP conference 2009 Bangladesh Institute of Development Studies

[B15] Population Reference Bureau (PRB) Globally and locally, a rich-poor gap persists Washington, DC 2007

[B16] Gwatkin D Initial country-level information about socio-economic differences in health, nutrition, and population 2003

[B17] Ashford LS Davidson RG Yazbeck AS Designing health and population programs to reach the poor 2005 Agency for International Development under the BRIDGE project. Population Reference Bureau

[B18] Silvia P Rekha M Aslihan K Charles A Targeting poverty and gender inequality to improve maternal health 2010 International Center for Research for Women

[B19] Bandara A Emerging health issues in Asis and the Pacific: implications for public health policy Asia-Pacific Development Journal 2005 12 2

[B20] Zere E Tumusiime P Walker O Joses K Mwikisa C Mbeeli T inequalities in utilization of maternal health interventions in Namibia: implications for progress towards MDG 5 targets International Journal for Equity in Health 2010 9 16 10.1186/1475-9276-9-16 20540793 PMC2898738

[B21] Houweling TAJ Ronsmans C Campbell OMR Kunst AE Huge poor-rich inequalities in maternity care: an international comparative study of maternity and child care in developing countries Bulletin of the World Health Organization 2007 85 10 10.2471/BLT.06.038588 PMC2636501 18038055

[B22] Zere E Moeti M Kirigia J Mwase T Kataika E Equity in health and healthcare in Malawi: analysis of trends BMC Public Health 2007 7 78 10.1186/1471-2458-7-78 17504530 PMC1884146

[B23] Pathak PK Singh A Subramanian SV Economic inequalities in maternal health care: prenatal care and skilled birth attendance in India, 1992-2006 PLoS ONE 2008 5 10 e13593 10.1371/journal.pone.0013593 PMC2965095 21048964

[B24] Praveen KP Rich-poor gap in utilization of delivery care services in India, 1992-2005 2006 International Institute for Population Sciences (IIPS), Govandi Station Road, Deonar, Mumbai-400088, India

[B25] Collin S Anwar I Rosmans C A decade of inequality in maternity care: antenatal care, professional attendance at delivery, and caesarean section in Bangladesh (1991-2004) International Journal of Equity in Health 2009 6 9 10.1186/1475-9276-6-9 PMC2014749 17760962

[B26] Ministry of Health, Government of Vanuatu Vanuatu Multiple Indicator Cluster Survey 2007, Final Report 2008 Port Vila, Vanuatu

[B27] Filmer D Pritchett LH Estimating wealth effects without expenditure data-or tears: an application to educational enrollments in states of India Demography 2001 38 115 132 11227840 10.1353/dem.2001.0003

[B28] Bloom SS Lippeveld T Wypij D Does antenatal care make a difference to safe delivery? A study in urban Uttar Pradesh, India Health Policy and Planning 1999 14 1 38 48 10.1093/heapol/14.1.38 10351468

[B29] Chakraborty N Islam MA Chowdhury RI Bari W Utilization of postnatal care in Bangladesh: evidence from a longitudinal study Health and Social Care in Community 2002 10 6 492 502 10.1046/j.1365-2524.2002.00389.x 12485137

[B30] Elo TI Utilization of maternal health-care services in Peru: the role of women's education Health Transition Review 1992 2 49 69 10148665

[B31] Rahman M Modes of delivery assistance in Bangladesh Tanzanian Journal of Health Research 2008 10 4 246 256 10.4314/thrb.v10i4.45081 19402587

[B32] Wagstaff A Paci P Van Doorslaer E On the measurement of inequalities in health Social Science and Medicine 1991 33 5 545 557 10.1016/0277-9536(91)90212-U 1962226

[B33] Chan YH Biostatistics: logistic regression analysis Singapore Med J 2004 45 149 15094982

[B34] Houweling TAJ Ronsmans C Campbell OMR Kunst AE Huge poor-rich inequalities in maternity care: an international comparative study of maternity and child care in developing countries Bulletin of the World Health Organization 2007 85 10 10.2471/BLT.06.038588 PMC2636501 18038055

[B35] Zere E Moeti M Kirigia J Mwase T Kataika E Equity in health and healthcare in Malawi: analysis of trends BMC Public Health 2007 7 78 10.1186/1471-2458-7-78 17504530 PMC1884146

[B36] Ministry of Health, the Republic of Vanuatu (MOH) National Health Accounts 2005, including Aus AID technical assistance would raise the donor share to 24 per cent of total health spending, and 30 per cent of public spending 2007 MOH, Vanuatu

[B37] Overbosch GB Nsowah-Nuamah NNN Van den Boom GJM Mamnyag L Determinant's of antenatal care use in Ghana Journal of African Economies 2004 13 277 301

[B38] Chakraborty N Islam MA Chowdhury RI Bari W Akhter HH Determinants of the use of maternal health services in rural Bangladesh Health Promotion International 2003 18 327 337 10.1093/heapro/dag414 14695364

[B39] Cochrane SH O'Hara DJ Leslie J The effects of education on health: a background study for world development report 1980 World Bank, Washington, D.C.

[B40] McDonagh M Is antenatal care effective in reducing maternal morbidity and mortality? Health Policy and Planning 1996 11 1 1 15 10.1093/heapol/11.1.1 10155875

[B41] Garenne M Mbaye K Bah MD Correa P Risk factors for maternal mortality: a case control study in Dakar hospitals (Senegal) African Journal of Reproductive Health 1997 1 14 24 10.2307/3583271 10214399

[B42] Hartfield VJ Maternal mortality in Nigeria compared with earlier international experience International Journal of Gynecology and Obstetrics 1980 18 70 75 6106608 10.1002/j.1879-3479.1980.tb00246.x

